# Lung Cancer and Interstitial Lung Diseases

**DOI:** 10.3390/cancers16162837

**Published:** 2024-08-13

**Authors:** Fotios Drakopanagiotakis, Ekaterina Krauss, Ira Michailidou, Vasileios Drosos, Stavros Anevlavis, Andreas Günther, Paschalis Steiropoulos

**Affiliations:** 1 Department of Pneumonology, Medical School, Democritus University of Thrace, 68100 Alexandroupolis, Greece; fdrakopanagiotakis@gmail.com (F.D.); anevlavis@yahoo.com (S.A.); 2 European IPF Registry & Biobank (eurIPFreg/Bank), 35394 Giessen, Germany; ekaterina.krauss@innere.med.uni-giessen.de (E.K.); andreas.guenther@innere.med.uni-giessen.de (A.G.); 3 Center for Interstitial and Rare Lung Diseases, Universities of Giessen and Marburg Lung Center (UGMLC), Member of the German Center for Lung Research (DZL), 35394 Giessen, Germany; 4 Department of Pneumonology, General Anti-Cancer Oncological Hospital, Agios Savvas, 11522 Athens, Greece; gataplan@gmail.com; 5 Department of Thoracic and Cardiovascular Surgery, University Hospital Würzburg, 97070 Würzburg, Germany; vedrosos@gmail.com; 6 Agaplesion Lung Clinic, 35753 Greifenstein, Germany; 7 Cardio-Pulmonary Institute (CPI), EXC 2026, Project ID: 390649896, Justus-Liebig University Giessen, 35394 Giessen, Germany

**Keywords:** interstitial lung disease, lung cancer, IPF

## Abstract

**Simple Summary:**

Lung cancer is one of the leading causes of cancer-related mortality worldwide. There is proof that interstitial lung disease and lung cancer interact and influence patient outcomes, treatment approaches, and the course of the disease. Common risk factors for both illnesses include smoking, exposure to the environment, and genetic predispositions. When interstitial lung disease is present, lung cancer management is complicated both diagnostically and therapeutically. These challenges include trouble interpreting radiological results and a higher risk of treatment-related toxicities, such as acute exacerbation following surgery and pneumonitis following radiation therapy and immunotherapy. Furthermore, the evidence-based treatment choices for patients with ILDs and lung cancer are still restricted.

**Abstract:**

Lung cancer continues to be one of the leading causes of cancer-related death worldwide. There is evidence of a complex interplay between lung cancer and interstitial lung disease (ILD), affecting disease progression, management strategies, and patient outcomes. Both conditions develop as the result of common risk factors such as smoking, environmental exposures, and genetic predispositions. The presence of ILD poses diagnostic and therapeutic challenges in lung cancer management, including difficulties in interpreting radiological findings and increased susceptibility to treatment-related toxicities, such as acute exacerbation of ILD after surgery and pneumonitis after radiation therapy and immunotherapy. Moreover, due to the lack of large, phase III randomized controlled trials, the evidence-based therapeutic options for patients with ILDs and lung cancer remain limited. Antifibrotic treatment may help prevent pulmonary toxicity due to lung cancer treatment, but its effect is still unclear. Emerging diagnostic modalities and biomarkers and optimizing personalized treatment strategies are essential to improve outcomes in this patient population.

## 1. Introduction

Lung cancer and interstitial lung diseases (ILD) share distinct biological pathways, even though the precise genetic and cellular mechanisms remain incompletely understood. ILDs can progress to pulmonary fibrosis, which is characterized by progressive scarring and lung tissue thickening, which can lead to impaired lung function [1]. Numerous signaling pathways and microenvironments have been identified as disruptors of tissue architecture, contributing to dysfunction in both conditions and leading to loss of lung function, impaired gas exchange, and respiratory symptoms such as dyspnea. Despite these commonalities, lung tumorigenesis and fibrosis are characterized by highly heterogeneous behaviors, emphasizing the need for personalized therapeutic approaches tailored to individual patients.

As an example, one of the most progressive forms of ILD, idiopathic pulmonary fibrosis (IPF), is influenced by genetic predisposition, aging, and environmental factors. Current evidence indicates that in genetically predisposed individuals, especially those over 60 years of age, environmental exposures lead to alterations in lung epithelium, initiating aberrant cellular activation and localized expansion of fibroblasts, ultimately resulting in fibrotic remodeling, loss of lung architecture, and functional deterioration, with the escalating mechanical stiffness perpetuating the fibrotic response through cell-autonomous and matrix-dependent processes [2].

Lung cancer is characterized by the uncontrolled growth of malignant cells in the lungs by activating various signaling pathways [3,4]. Although lung cancer and pulmonary fibrosis are distinct clinical conditions, evidence suggests shared pathomechanisms, aggravating factors, and signaling pathways between them. Indeed, the coexistence of pulmonary fibrosis and lung cancer is not uncommon [5,6]. The existence of IPF itself causes a 7% to 20% increased risk of lung cancer development [7]. Also, the prevalence of lung cancer is significantly higher in the ‘combined IPF and emphysema’ (CPFE) cohort as compared with patients with pulmonary fibrosis [8].

Moreover, lung transplant patients with IPF exhibit higher rates of lung cancer, suggesting shared molecular connections between the two diseases [9]. Such shared molecular pathways of established lung cancer and pulmonary fibrosis are the epithelial–mesenchymal transition (EMT), mesenchymal activation, and SFTPA mutation [10].

Also, lung cancer manifests in the peripheral regions of the lungs, where especially fibrotic changes typical of usual interstitial pneumonia (UIP) are prevalent, and adenocarcinoma and squamous cell carcinoma are the predominant types observed in individuals with IPF [11]. In more detail, pulmonary fibrosis and lung cancer share common risk factors, such as smoking, occupational/environmental exposures (e.g., asbestos, silica), and genetic predisposition. These risk factors may lead to developing both conditions in the same individual, significantly impacting disease survival [8].

The global burden of lung cancer and ILDs is increasing [6,12,13]. Since the risk of lung cancer in patients with pulmonary fibrosis is increased, there is growing interest in instituting screening and surveillance programs for this population. The presence of pulmonary fibrosis in individuals with lung cancer has clinical implications, influencing both treatment decisions and prognosis [14]. The compromised lung function associated with pulmonary fibrosis may limit specific treatment options, and the overall prognosis for individuals with both conditions may be poorer compared to those with lung cancer alone. Early detection of lung cancer is anticipated to facilitate more effective treatment and improve outcomes. This emphasizes the importance of understanding the interplay between pulmonary fibrosis and lung cancer for better management and outcomes in affected individuals [6,15].

As research unravels both conditions’ intricate molecular and cellular landscapes, the prospect of identifying converging points in the pathogenic processes holds promise for innovative and more effective therapeutic interventions. Integrating insights from the research of cancer and fibrosis not only enhances our understanding of these diseases but also offers a potential roadmap for developing targeted therapies that address the unique challenges posed by the heterogeneity observed in lung tumorigenesis and fibrogenesis. Recognizing the commonalities in the underlying pathways between cancer and pulmonary fibrosis opens avenues for the development of novel therapeutic strategies and personalized approaches and may prove essential in optimizing treatment outcomes of individuals with lung cancer and fibrosis.

## 2. Pathophysiology

Specific pathologic mechanisms and factors have been proposed to contribute to the relationship between ILDs and lung cancer. As indicated by genetic alterations in specific genes and signaling pathways, shared genetic and molecular pathways may contribute to developing both pulmonary fibrosis and lung cancer [2,6,16]. This shared susceptibility involves such genetic factors as aberrations in essential genes like TGF-β and p53, with evidence suggesting that specific genetic factors related to lung development, tissue repair, and cell cycle regulation, such as mutations in relevant genes, may play roles in the development of both conditions [17].

Specific gene mutations, including those involving microsatellite instability, fragile histidine triads, the oncogene p53, and loss of heterozygosity, are observed in many IPF cases, particularly in the characteristic peripheral lung regions with honeycombing [18]. Mutations associated with cancer, such as those affecting telomere shortening and telomerase expression, are also found in familial IPF, suggesting shared genetic factors [19]. Jan kinase and SFTP mutations were found in families with PF and lung cancer.

Chronic inflammation is a common feature in pulmonary fibrosis and tumor development and is associated with the fibrotic process in some ILDs [6,20]. The inflammatory microenvironment may create conditions that promote the initiation and progression of cancer cells in the lungs [21]. Furthermore, the fibrotic changes in pulmonary fibrosis involve excessive collagen deposition and other extracellular matrix components, leading to tissue remodeling and scarring [22]. This altered lung architecture and increased stiffness may create a microenvironment that promotes cancer cell growth [23,24] and may involve modifications in the extracellular matrix and cytokine profiles [10,25].

Tyrosine kinases, integral to multiple signaling pathways regulating cell growth, differentiation, adhesion, motility, and cell death, are normally controlled by transmembrane receptors and ligands. However, aberrant kinase activities have been linked to various cancers’ development, progression, and metastasis and some types of ILD [26]. It is in line with this note that nintedanib, a triple kinase inhibitor, is effective in both lung fibrosis and lung cancer and is authorized for both conditions [26]. Further, oncogene hypomethylation and tumor suppressor genes’ methylation, which are involved in the pathogenesis of many tumors, are also present in patients with IPF. Recent data show reciprocal changes in global methylation patterns between IPF and lung cancers and hypermethylation of the CD90/Thy-1 promoter region in IPF, contributing to the loss of the glycoprotein Thy-1, associated with invasive cancer behaviors and the transition from fibroblasts to myofibroblasts [27].

Fibrosis is intricately linked to the persistent action of transforming growth factor (TGF)-β, a regulator of various intracellular mediators and pathways associated with cell growth, organ development, the immune system, metastasis, and cancer progression. The “TGF-β paradox” is observed, according to which TGF-β promotes cellular growth in cancer cells but has the opposite effect in benign cells [28]. This results from the extracellular signal-regulated kinase (ERK) pathway activation in malignant cells and its inactivation in non-cancer cells.

TGF-β, VEGF, PDGF, and FGF are implicated in both lung cancer and lung fibrosis, with VEGF potentially promoting cell survival and proliferation through ERK1/2 and PI3K activation and VEGF mRNA being elevated levels in IPF patient endothelial progenitor cells [29]. The downregulation of TGF-β receptors is a pivotal event leading to changes in cellular behavior, with low receptor levels promoting metastasis and cancer progression and playing a crucial role in early carcinogenesis [30]. Lung cancer in individuals with pulmonary fibrosis tends to be more aggressive, with TGF-β playing a critical role by being produced by both pulmonary fibrosis-associated fibroblasts and cancer-derived epithelial cells, promoting myofibroblast recruitment at cancer margins, safeguarding them from apoptosis, and facilitating their invasion through basement membranes and the process of EMT [31].

Furthermore, elevated expression of programmed cell death-ligand 1 (PD-L1) is seen in both lung cancer and IPF, where PD-L1, a cell surface protein, interacts with the PD1 receptor on T-cells, leading to immune response suppression and contributing to the pathogenesis of these diseases [32].

Additionally, the MET signaling pathway (a receptor tyrosine kinase whose ligand is hepatocyte growth factor), a crucial regulator of cell growth and proliferation activated in response to hypoxia, plays a significant role in both IPF and lung cancer [33]. Activation of the MET pathway promotes increased cell proliferation, tumor growth, and the expression of genes related to cell proliferation; the upregulation of the MET signaling pathway occurs in fibroblasts and myofibroblasts, contributing to the excessive collagen deposition and tissue fibrosis characteristic of the disease [34].

Pulmonary fibrosis is linked to cellular senescence, a state characterized by the secretion of pro-inflammatory factors that promote inflammation and tissue remodeling, potentially contributing to the development of cancer through the senescence-associated secretory phenotype (SASP) and the creation of a microenvironment supportive of cancer growth [35].

In both cancer and pulmonary fibrosis (particularly IPF), bronchiolar basal cells express molecules, including fascin, laminin, and heat shock protein 27, which are associated with cell migration and invasion, contributing to the invasive front of tumors and expressed in epithelial cells around fibroblast foci [36]. Additionally, matrix metalloproteases and integrins, known for their role in cell invasion, are strongly linked to the development of stem cell-like properties in cancer cells and, in the context of IPF, promote the initiation, maintenance, and resolution of tissue fibrosis, with clinical trials investigating inhibitors such as the humanized antibody STX-100 and specific antibodies against αvβ6 [37].

EMT is a process where epithelial cells undergo changes to become more mesenchymal, and this is implicated in both fibrosis and cancer. The transition of cells from an epithelial to a mesenchymal phenotype can promote tissue remodeling and contribute to cancer progression. EMT is implicated in fibrosis and cancer metastasis. EMT may contribute to tissue remodeling in the lung and create a microenvironment supporting cancer growth [38].

Circulating and cell-free DNA, as well as abnormal expression levels of mRNA, are considered diagnostic and prognostic biomarkers for both cancer and IPF. The aberrant expression of specific short non-protein-coding RNAs in IPF influences genes associated with fibrosis, ECM regulation, EMT induction, and apoptosis, potentially contributing to functional deterioration in patients with pulmonary fibrosis [39,40].

Intercellular channels formed by connexins (Cxs) are crucial in cells’ metabolic and electrical coupling. The most abundant Cx on fibroblast membranes is Cx43, which contributes to tissue repair and wound healing; however, the expression of Cx43 in primary lung fibroblasts of IPF patients is reduced, leading to limited intercellular communication. This reflects common defects in contact inhibition and uncontrolled proliferation seen in both IPF and cancer cells [41].

The main pathologic mechanisms contributing to the development of lung cancer in pulmonary fibrosis are summarized in Table 1.

## 3. Epidemiology of Lung Cancer in ILD

IPF is an established risk factor for lung cancer. An increased risk of lung cancer in patients with other ILDs has also been reported, although data regarding other ILDs are limited. Even the finding of ILAs in CT screening has been associated with an increased risk of 33% of lung cancer [42]. Other studies have suggested that the HR of lung cancer diagnosis in patients with ILAs is as high as 2.77 [43]. Patients with increased risk for lung cancer in ILDs are male patients of older age, decreased lung function with a decline in FVC > 10% per year, low DLCO, and smoking [44,45]. The risk of lung cancer in patients with combined pulmonary fibrosis and emphysema is nine times higher compared to subjects without underlying disease [46,47].

Survival outcomes vary based on the ILD subtype, with lung cancer survival being poorer in patients with IPF compared to those with nonspecific interstitial pneumonia (NSIP) or cryptogenic organizing pneumonia (COP) [48,49,50].

Lung cancer in patients with UIP pathological pattern is mainly found in the subpleural areas of the lower lobes (75.5%), a finding consistent with the typical distribution pattern of UIP [51,52]. Squamous cell carcinoma was the most common cancer type (62.8%) in this group [52]. In contrast, in patients with ILD and non-UIP histology, lung cancer primarily occurred in the upper lobes (68.1%). Adenocarcinoma was the predominant cancer type and was found in more than half of this group of patients [51]. In non-IPF lung cancer cases, levels of Krebs von Lungen factor 6 (KL6) have been associated with prognosis [53]. Data regarding the prevalence, histology, and location of lung cancer in patients with ILDs are shown in Table 2.

An important aspect regarding the epidemiology of lung cancer in patients with ILDs is the establishment of screening programs for lung cancer with low-dose CT (LDCT). LDCT has significantly changed the staging of lung cancer, increased the detection of early-stage cancers, and reduced late-stage diagnoses [54]. Moreover, LDCT in the setting of lung cancer screening has led to an increased diagnosis of ILAs. Specific guidelines regarding the management of ILAs as an incidental finding have been published [54]. As described before, ILAs are associated with an increased risk of developing lung cancer. Moreover, LDCTs in the setting of a lung cancer screening program are related to the finding of true ILD. In a screening program of 6650 participants, ILDs were first diagnosed in 0.8%, including IPF [55]. Early findings of ILDs can not only lead to early initiation of appropriate treatment for the ILDs but also identify patients with concomitant lung cancer in an earlier stage, thus increasing the chance of definitive surgical treatment for these patients [56].

### 3.1. IPF

An estimated 17.5-fold greater prevalence of NSCLC among IPF patients compared to the general population has been reported in a large Korean database, even after adjustment for age, smoking history, and sex [57].

A study of a large Medicare database of approximately 55,000 patients with NSCLC reported a prevalence of IPF of 1.6% [58]. Studies from various regions consistently report elevated lung cancer prevalence in IPF patients compared to the general population, with rates ranging from 2.7% to 48%, with a median prevalence of 11.6% in European cohorts of patients with IPF and 15.3% in Asian cohorts [59]. In Korea, the prevalence of lung cancer in IPF was reported to be 6.4% [60]. A recently published European large multicenter study of 3178 patients with IPF reported the development of lung cancer in 10.2% of the patients [52].

For IPF patients, the cumulative incidence of lung cancer rises significantly, ranging from 1.1% at one year, 8.7% at three years, 15.9% at five years, and 31.1% at ten years of follow-up [44]. A longitudinal cohort study from Korea showcased a cumulative NSCLC incidence of 3.3%, 15.4%, and 54.7% at 1, 5, and 10 years post-IPF diagnosis, respectively [61]. In a large European study, the incidence of lung cancer in IPF was 14.1 and 26.6% among patients who were alive upon completion of the three-year and ten-year follow-up, respectively [52]. The incidence of lung cancer in IPF cases is considerably higher compared to the general population, with reported rates ranging from 3.34 to nearly 5 [62,63]. In a study of NSCLC in patients with ILD, lung cancer was manifested approximately 2.4 years following ILD diagnosis [64].

Among individuals with IPF, the most prevalent histological type is squamous carcinoma, followed by adenocarcinoma, contrary to the general population, in which adenocarcinoma is the most common histological type [60,65]. Mucinous adenocarcinoma has also been reported to have increased frequency in IPF [66]. Patients with IPF have a higher frequency of lung cancer occurrence in the lower lobes compared to other ILD patients and to the general population, who have a propensity for the upper lobes [52,65].

The prognosis for NSCLC patients with concurrent IPF is poorer than for those with either condition alone, persisting even when adjusting for baseline lung function, with a 5-year survival of 14.5% for lung cancer patients with IPF compared to 30.1% for those without [67]. This difference includes stages I-III of NSCLC, while in stage IV NSCLC and SCLC, the survival difference is similarly low in IPF vs. non-IPF categories [67,68,69]. Reasons for the worse survival of these patients include poor pulmonary status and comorbidities, which exclude many patients with ILDs from surgery as a therapeutic option, treatment complications, such as acute exacerbations perioperatively or due to systemic therapy or pneumonitis due to immunotherapy, the possible detrimental effects of radiation therapy and diagnosis of lung cancer in a more advanced stage, since symptoms of NSCLC and ILDs may overlap [70].

### 3.2. Connective Tissue Disease (CTD)-Related ILD

Patients with connective tissue disease (CTD)-related ILD usually present with different clinical characteristics as patients with IPF: these patients are primarily women and younger than the patients with IPF [43]. Moreover, the histological pattern of NSIP and not only UIP can be found in many patients with CTD-ILD. Autoimmunity and inflammation play an essential role in the pathogenesis of the CTD. The likelihood of developing lung cancer is almost two times higher in patients with CTD-related ILD compared to ILD patients without CTD. The relative risk is increased from 3.5 to 7.3 for any ILD subtype [71]. Lung cancer incidence has been reported to be higher in younger patients: the incidence of lung cancer in men with CTD-ILD aged 40–49 years was 3.2 times higher compared to men with ILD of the same age, and the incidence in women with CTD-ILD aged 50–59 years was 2.8 times higher [71]. Mortality differences have also been reported, with the all-cause mortality rate being higher in older CTD-ILD patients (50 to 79 years old) than in those ILD-only, especially in women [71].

Data from a US registry suggest that in individuals with ILD and conditions like rheumatoid arthritis, polymyositis/dermatomyositis, or systemic sclerosis, the risk was significantly increased, reaching up to 4.95 times higher as compared to the general population [62]. The prevalence of lung cancer in CTD-ILD was 1.9% [62]. A Japanese study has reported a prevalence of 9%, with risk factors being male gender, heavy smoking, older age, presence of emphysema, a UIP pattern, and non-receiving immunosuppressive therapy [45].

Systemic sclerosis and dermatomyositis/polymyositis with ILD are associated with lung cancer risk: systemic sclerosis was associated with an incidence of 11.1%. In comparison, dermatomyositis/polymyositis and rheumatoid arthritis were associated with an incidence of 4.4% [72]. In patients with systemic sclerosis, lung cancer has been reported as the most common cancer, with an incidence ranging from 4.9–5.7% [73].

In a recently published study of 51,899 patients with newly diagnosed rheumatoid arthritis, the hazard ratio of developing lung cancer in 4.5 years of follow-up was 1.39. This correlated to male gender and smoking but, surprisingly, not to the presence of ILD [74]. However, the presence of ILD is a significant risk factor for mortality in patients with rheumatoid arthritis who are also diagnosed with lung cancer: The estimated mortality of lung cancer in patients with chronic ILD with a UIP pattern was five times higher than the general population in a study of 2702 patients with rheumatoid arthritis [75].

It seems that chronic inflammation, combined with the detrimental effect of smoking, plays an essential role in the development of lung cancer in patients with CTD-ILDs [62]

### 3.3. Hypersensitivity Pneumonitis (HP)

HP is caused by exposure to organic antigens. Chronic HP usually presents as progressive pulmonary fibrosis. Differential diagnosis between chronic HP and IPF can be challenging. Kuramochi et al. reported a 10.6% prevalence of lung cancer in HP, with the most common histological type being squamous cell carcinoma in basal, peripheral lung lesions. Interestingly, lung cancer patients with chronic HP had a UIP pathology, suggesting that progressive pulmonary fibrosis and parenchyma distortion due to fibrosis, irrespective of its etiology, contribute to the development of lung cancer [76]. Other studies have reported that chronic HP contributed to 5.8% of all non-IPF ILD lung cancer cases [53]. In a prospective database of patients with chronic HP, however, followed up for 32 months, no lung cancer cases were observed [77].

### 3.4. Post-Infectious Fibrosis

In the setting of the COVID-19 pandemic, a pulmonary fibrosis ‘tsunami’ was expected to follow the COVID-19 ‘earthquake’ [78]. Since fibrosis is associated with an increased risk of lung cancer, an increased incidence of lung cancer might also occur. Luckily, such a ‘tsunami’ did not happen, and an increased incidence of lung fibrosis post-Covid does not seem to be a significant clinical problem at the moment. However, since millions of people were affected by the virus, caution is required to exclude such a relationship in the future [79].

**Table 2:** Prevalence, histology, and location of lung cancer in patients with ILDs, based on refs. [45,52,59,62,76,77].

ILD	Prevalence of LC	Most Common LC Histology	Location
IPF	Median 11.6% in European cohorts and 15.3% in Asian cohorts	Squamous cell carcinoma	Lower lobes, fibrotic areas
CTD-ILD	1.9–9%	Adenocarcinoma	Peripheral lung lesions, equal distribution in upper and lower lobes
Hypersensitivity pneumonitis	0–10.6%	Squamous cell carcinoma	Peripheral lung lesions, equal distribution in upper and lower lobes

LC: lung cancer; IPF: idiopathic pulmonary fibrosis; CTD-ILD: connective tissue disease-associated ILD.

## 4. Diagnostic Approach to the Patients with ILD and Suspected Lung Cancer

**Figure 1:**
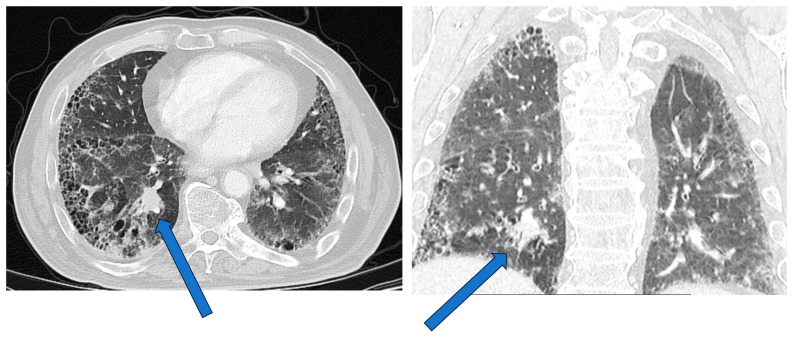
Lung cancer in a patient with combined pulmonary fibrosis and emphysema. The tumor is located in the right lower lobe (arrows).

In individuals with ILD, diagnosis of lung cancer can be difficult since clinical symptoms and functional changes can be attributed to the ILD until lung cancer is suspected. Moreover, suspicious, subtle changes in chest CT may be recognized late and retrospectively due to the existing fibrotic lung alterations (Figure 1) [44,52]. Careful comparison of CTs to recognize new solitary nodules is important. Since ILDs (particularly IPF) are a risk factor for the development of lung cancer development, annual HRCTs for follow-up of the IPF and eventual development of pulmonary nodules should be strongly considered in these patients [80]. New pulmonary nodules should be further evaluated according to Fleischner criteria’s high-risk group [80,81]. It is important to consider that the tumor size may be underestimated by 10% in patients with ILDs [73]. Additionally, evaluation of mediastinal lymph nodes with CT can be difficult in patients with ILD since reactive mediastinal lymph node enlargement is common in these patients, reducing the specificity of this method for detecting lung cancer [82,83].

Most experts would agree that PET-CT can help to identify the malignant character of nodules with a size above 8 mm and provide information for possible extra thoracic lung cancer involvement, which is accessible to biopsy [84,85,86]. Tissue sampling of suspicious mediastinal lymph nodes can be made using endobronchial ultrasound-guided transbronchial needle biopsy (EBUS-TBNB) and peripheral lesions with radial EBUS [87]. The latter can be challenging due to fibrotic changes in the surrounding lung [87,88].

CT-guided transthoracic needle biopsy is an alternative, technically more difficult in patients with ILD when the nodule is located in the lower lobes due to motion artifacts and non-diagnostic results due to sampling of fibrotic areas [89]. Pneumothorax may be a severe complication of CT-guided biopsy and is often associated with poor prognosis in patients with ILDs [90]. Placement of a tube drainage may be required in 10% of the patients. Factors associated with an increased risk of pneumothorax include lesion size <3 cm, a target lesion depth exceeding 1 cm, longer procedure duration, and honeycombing or emphysema along the needle’s path [89,91]. Trying to avoid inserting the needle through honeycomb lesions might reduce the risk of pneumothorax, but this cannot be easily achieved. CT-guided biopsy in the prone position is associated with a significantly reduced risk of pneumothorax requiring tube drainage [91,92]. Immediate manual aspiration of pneumothorax has been reported to be associated with reduced risk of chest tube drainage placement, even for large pneumothoraxes [91].

The above approach might be more appropriate for patients with mild to medium lung functional impairment [70]. The approach to patients with end-stage fibrotic ILD, severely debilitated due to respiratory failure, might be more conservative since therapeutic options in these patients for lung cancer may be limited. A ‘liquid biopsy’ aiming to disclose driver mutations and identify individual treatment in debilitated patients may also be considered. In all cases, shared decision-making should be performed with the patients [70].

Limitations of the diagnostic modalities in patients with ILDs suspected of having lung cancer are presented in Table 3.

## 5. Treatment of NSCLC in Patients with ILDs

### 5.1. Surgical Therapy

Surgical resections in patients with ILD remain challenging, due to the fact that patients with ILD who are considered for surgical treatment of their malignancy often have an impaired lung function, advanced age, and many comorbidities. Moreover, a surgical procedure is a risk factor for postoperative AE-ILD, which increases the perioperative mortality [93]. As a result, ILD per se is a risk factor for increased morbidity and mortality after lung resection for lung cancer [94]. A retrospective cohort analysis of 128,723 patients who underwent lung surgery for NSCLC, among them 1873 with ILD, showed a postoperative mortality of 5.1% in patients with ILD vs. 1.2% in patients without ILD, ARDS 1.9% in ILD vs. 0.5% without ILD and of composite morbidity and mortality of 13.2% in ILD vs. 7.4% without ILD [94]. ILD remained a predictor of mortality, even after adjustment for lung function, comorbidities, and extent of resection [94].

Kumar et al. [95] reported an incidence of post-operative AE-ILD of 21%, with a mortality of 80%. The extent of surgical resection was also associated with mortality [95]. In a study of Park et al., the reported incidence of acute complications (ARDS and ALI) was 28% in 100 patients, with an overall operative mortality of 14%. Pre-existing comorbidities and reduced DLCO were associated with increased risk of AE-ILD in univariate analysis [96]. In a large, retrospective study from Japan, Sato et al. [97] analyzed data of 1763 patients with ILD who underwent pulmonary resections for lung cancer. The study showed an incidence of AE of 9.3% (164 patients) with a mortality rate of 43.9%. AE-ILD was the main cause of 30-day mortality, accounting for 72% of deaths. Risk factors for AE-ILD were male sex and history of previous AE-ILD. A UIP pattern in HRCT and an FVC value of <80% predicted were also identified as risk factors for AE-ILD [97]. Preoperative steroid use was associated with a 2.86 times increased ratio of AE-ILD. KL-6 serum levels above 1000 U/mL correlated to increased risk of AE-ILD (OR 2.14) [97]. Patients with CPFE have been reported to have an increased mortality comparable to the patients with emphysema alone. The incidence of AE-ILD was lower as in patients with IPF alone [98].

Min Seo Ki et al. [99] demonstrated in a propensity score matching study of 104 patients that long-term prognosis is affected negatively in ILD (5-year survival rate 66% vs. 78.8%, *p* = 0.007). However, there was no difference between groups in postoperative mortality [99]. Moreover, mortality was significantly higher in patients with a stage III ILD-GAP score [99]. Ueno et al. also reported a correlation of GAP score with mortality [100]. In the same study, the degree of fibrosis in HRCT, increased BMI, and a higher CRP were independently associated with mortality [100].

Regarding the resection range, most studies report an increased risk of complications, including AE-ILD, with more extensive surgery [97]. Compared to a wedge resection, performance of lobectomy or segmentectomy had an OR of 3.83 and performance of a pneumonectomy or bilobectomy had an OR of 5.7 of AE-ILD [97]. Huang et al. also showed that patients who underwent lobectomy or lobectomy had a higher range of complications compared to the patients who underwent sub-lobar resections [93]. In this study, the AE rate was 15.4%, with a mortality of 9.0%. In the multivariate analysis, however, age, comorbidities, and blood loss but not the extent of surgery were the risk factors for 90-day complications [93]. Tsutani et al. [101], in a study of 107 patients with stage I NSCLC, showed no difference in overall survival between patients who underwent lobectomy and sub-lobar resections. However, the risk of a higher locoregional recovery by very limited resections cannot be ignored. Regarding sub-lobar resections, Motono et al. showed that in patients with ILD and stage IA NSCLC, wedge resection is a poor prognostic factor and that a segmentectomy seems a better option [102]. Lobectomy is currently compared to segmentectomy and wedge resection in phase III randomized controlled trials examining the clinical effectiveness of each method for early NSCLC with IPF, with the primary endpoint being the overall survival [103]: a planned total of 430 patients will be enrolled from 50 institutions over five years [103]. In a recent meta-analysis of 2202 patients with UIP pattern, the overall incidence of AE of UIP postoperatively was 14.6%. Patients who underwent a sub-lobar resection were at significantly lower risk of postoperative AE (OR 0.521) [104].

Apart from the extent of the surgical resection, the location of the resected lobe is another important factor regarding the postoperative course of patients with ILDs: Fukui et al. [105] studied the difference between upper and lower lobectomy and concluded that there is no statistical difference in mortality and morbidity. This remained after adjusting for the simultaneous presence of emphysema. However, upper lobectomy had a more significant impact on postoperative respiratory function [105].

Repeated surgery for lung cancer in patients with ILD is rare and highly challenging. Sato et al. showed in a small sample study (of 13 patients) that repeated surgery was associated with a high percentage of AE-IPF. Still, it could be beneficial in carefully selected patients [106].

An important aspect is the prevention of perioperative AE-ILD. Pirfenidone is an antifibrotic agent licensed for the treatment of patients with IPF. In a small phase II trial, administering pirfenidone perioperatively was safe and had promising results in preventing AE-ILD [107]. In a retrospective study of 100 patients with IPF who were surgically treated for lung cancer, patients treated with pirfenidone had fewer AE-IPF compared to the patients without antifibrotic treatment [108]. A prospective phase III study examining the effect of pirfenidone in preventing AE-IPF and survival is running [109].

### 5.2. Radiation Therapy

Radiation of the lesions in the lung is an integral part of the therapy in lung cancer. In general, it is indicated for patients with early-stage NSCLC, who are not surgical candidates due to poor lung function or comorbidities, in cases of R1 surgical resection and locally advanced NSCLC combined with chemotherapy [110,111]. In SCLC, lung radiation therapy is indicated in limited disease [112]. Moreover, lung radiation therapy is indicated in urgent situations, such as superior vena cava syndrome [111]. Stereotactic body radiation therapy (SBRT) is the treatment of choice for non-surgical candidates with early-stage disease due to the reduced risk of induced side effects [111,113]. Radiation therapy, particularly when applied for locally advanced disease, affects larger lung fields and requires higher radiation doses. A well-known side-effect of radiation therapy is radiation pneumonitis [114]. For this reason, patients with ILDs have been excluded from clinical trials of radiation therapy in patients with lung cancer [114].

Retrospective data suggest that the frequency of radiation pneumonitis in patients with ILDs is significantly higher than patients without ILD: A study involving 537 Stage I lung cancer patients treated with SBRT, including 39 patients with ILD-related imaging features, revealed that ILD patients had significantly higher rates of grade ≥2 (20.5% vs. 5.8%) and grade ≥3 (10.3% vs. 1.0%) radiation pneumonitis compared to non-ILD patients [115]. Moreover, ILD patients exhibited more extensive radiation pneumonitis beyond the radiotherapy treatment fields. Patients with stage I lung cancer and a prior diagnosis of ILD had a significantly higher rate of severe radiation pneumonitis compared to the overall group [116]. Specifically, 32% of ILD patients experienced grade ≥3 radiation pneumonitis, and 21% had grade 5 radiation pneumonitis [116]. In an analysis of 66 patients involving treatment with SBRT of primary and metastatic lung tumors, the presence of subclinical ILD was identified as the sole factor significantly linked to the occurrence of grade 2 to 5 radiation pneumonitis [117].

Preliminary data suggest that proton beam therapy (PBT) may be more advantageous than SBRT, particularly for patients with ILDs [118]. However, PBT is not widely available and large data series regarding its use in ILDs and lung cancer are missing.

A retrospective analysis of 87 patients with subclinical ILD and NSCLC who received conventionally fractionated radiotherapy also examined the impact of radiotherapy on ILD [119]. Most of the patients had stage III NSCLC and received sequential chemoradiotherapy. Grade ≥2 radiation pneumonitis occurred in 51.7% of patients, while more severe radiation pneumonitis was associated with concurrent chemoradiotherapy. The mean lung dose, the ILD involvement, and prior gemcitabine use were linked to increased risk of severe pneumonitis [119]. ILD involvement of >10% of lung field predicted acute development of radiation pneumonitis [120]. Increased SUV of the fibrotic lung in PET-CT has also been associated with increased frequency of radiation pneumonitis [121].

According to a systematic review trying to summarize the above aspects, the overall median incidence of grade ≥3 radiation pneumonitis in patients with ILDs undergoing radiation therapy for lung cancer was 19.7% (range 8–46%). Patients treated with particle beam therapy or stereotactic ablative radiotherapy had a lower incidence (median 12.5%) than those treated with conventional radical radiotherapy (31.8%). Grade 5 radiation pneumonitis occurred with a median rate of 11.9% (range: 0–60%). The existence of ILD independently predicted severe radiation pneumonitis. When idiopathic pulmonary fibrosis (IPF) or the usual interstitial pneumonia (UIP) pattern were present, severe radiation pneumonitis was more common than when these conditions were not [114].

Radiotherapy is not commonly used in patients with IPF due to its possible detrimental effects. According to a recently published retrospective, multicenter European study, only a small percentage of 12.5% of patients diagnosed with both IPF and lung cancer underwent radiotherapy [52]. However, physicians involved in the clinical care of patients with IPF and lung cancer would consider radiotherapy, particularly SBRT in carefully selected patients [70].

### 5.3. Percutaneous Ablation

Percutaneous image-guided ablation is a technique for treating small tumors in early NSCLC, with results like sub-lobar resection and SBRT [122]. It can be applied as radiofrequency, microwave, or cryoablation [122,123]. Due to the local effect, this approach might be of value in patients with ILDs [124], although data are scarce: mortality rates of 7.1% to 8.7% due to AE-ILD have been reported with a lung toxicity of 25% [125,126]. Complications, such as pneumothorax, bronchopleural fistula, and pneumonia have been reported [124,127].

### 5.4. Systemic Therapy for Lung Cancer in Patients with ILDs

#### 5.4.1. Chemotherapy

Chemotherapy plays a cardinal role in the treatment of patients with locally advanced and metastatic lung cancer [111,112]. Regarding NSCLC, histology and molecular markers are decisive for the appropriate treatment regimen selection. Before the introduction of immune checkpoint inhibitors (ICI), patients typically underwent first-line chemotherapy, often involving a platinum-based doublet regimen [128,129]. This regimen commonly included either carboplatin or cisplatin along with taxanes, etoposide, or pemetrexed. Patients with ILDs have been mostly excluded from phase III controlled randomized trials examining the effect of systemic therapy in lung cancer. This was due to the possible risk of AE-ILD and drug-induced pneumonitis in patients with ILDs. However, case series, registries, phase II trials, and a phase III trial (J-Sonic trial) have given insight into the effect of chemotherapy on patients with ILDs, particularly IPF [73,130]. Patients with IPF and SCLC have a significantly higher incidence of AE-IPF than patients with IPF and NSCLC after first-line treatment (31% vs. 63%) [131]. A recent meta-analysis has shown that AE was significantly more common in IPF than in non-IPF ILD after chemotherapy [132]. Increased FDG avidity in the contralateral lung on FDG PET/CT has been reported to be correlated with the risk of chemotherapy-related AE and may assist in identifying high-risk patients [133]. Certain medications increase the risk of pneumotoxicity and AE-ILD. A meta-analysis of 684 patients with ILD who underwent first-line chemotherapy for NSCLC showed a response rate of 43%. AE-ILD was approximately 8% in the context of chemotherapy, with lower rates (5%) observed in regimens containing nab-paclitaxel compared to other regimens (12%) [134]. For patients receiving docetaxel or gemcitabine, reported ILD exacerbation rates were 28% and 43%, respectively, while vinorelbine was not associated with AE-ILD in a small retrospective study [131]. Pemetrexed has shown increased toxicity in patients with IPF compared to those with other ILDs and significantly higher compared to patients without underlying ILD [135,136].

Experimental data suggest an inhibitory effect of pirfenidone on the progression of lung cancer [137,138,139,140,141]. A small Japanese study of 14 patients with IPF and NSCLC found that the combination of pirfenidone with paclitaxel or S-1 was safe, while no AE-IPF was seen in these patients [137].

The above data illustrate the effectiveness of first-line chemotherapy with platinum doublets in patients with NSCLC and ILD [134]. Although the incidence of AE-ILD was generally less than 10%, some patients died due to AE-ILD [134].

#### 5.4.2. Targeted Therapy

Targeted therapies, such as EGFR tyrosine kinase inhibitors or anaplastic lymphoma kinase inhibitors (ALK), have been associated with pneumotoxicity [138]. Some studies suggest that targeted therapies might be related to an increased risk of pneumotoxicity in patients with pre-existing ILD [139]. However, no large studies evaluating these treatments in patients with underlying ILD exist. ILD occurs in up to 5.2% of patients treated with EGFR TKIs for NSCLC, with a grade 3 or higher ILD affecting up to 2.2%. A high mortality in EGFR-TKI-induced ILD has been reported. In a recent meta-analysis, male sex, smoking history, and pre-existing ILDs have been reported as risk factors for EGFR-TKI-induced ILDs. Pre-existing ILD was associated with a six times increased risk of developing EGFR-TKI-induced ILD [140,141,142].

#### 5.4.3. Anti-VEGF Agents

Nintedanib is a tyrosine–kinase inhibitor that exhibits its antiangiogenic properties by targeting the vascular endothelial growth factor receptor (VEGF), fibroblast growth factor (FGF) receptor, and platelet-derived growth factor (PDGF) receptor. In combination with docetaxel, it is used as a second-line therapy in patients diagnosed with adenocarcinoma after failure of first-line chemotherapy [143]. Nintedanib has also been approved for the treatment of IPF, as it was shown that it slows down the progression of the disease and reduces the risk of acute exacerbation at a dose of 150 mg bid [144].

There were observations in patients with NSCLC and IPF who received nintedanib to treat the progression of IPF that a reduction of the size of cancerous lesions was achieved as well, while lung function was preserved. The doses used for treatment were 100 to 150 bid [145,146,147,148]. In an attempt to examine the optimal therapy for patients with lung cancer and IPF, a randomized phase 3 trial (J-SONIC) compared the efficacy and safety of nintedanib plus chemotherapy to chemotherapy alone [130]. In this study, the primary end-point, which was exacerbation-free survival, was not met, as there was no significant difference between the two groups. The combination of nintedanib with chemotherapy was well-tolerated and prevented FVC decline at 12 weeks. Overall response rate (ORR) was significantly better for nintedanib plus chemotherapy vs. chemotherapy in nonsquamous NSCLC [130]. Overall survival was also better in patients with nonsquamous NSCLC and patients with a stage I GAP (gender–age–physiology) [130].

The anti-VEGF antibody bevacizumab has been tested in patients with ILD and lung cancer and has been reported to prevent chemotherapy-induced AE-ILD [149,150].

Since ILD is the most severe complication of EGFR-TKIs, efforts to reduce the risk of pneumotoxicity are of particular importance: a recent meta-analysis examined the effect of anti-VEGF treatment, such as nintedanib, bevacizumab, and ramucirumab, on EGFR-TKI-induced ILD. The authors reported that combining EGFR-TKIs with anti-VEGF agents was associated with a significantly reduced incidence of ILD compared with EGFR-TKI monotherapy. Since nintedanib has been licensed for treating IPF, scleroderma-associated ILD, and progressive pulmonary fibrosis, the combination with EGFR-TKIs may have significant implications for patients with ILD and NSCLC [151].

Other reports suggest that nintedanib might reduce the pneumotoxicity of ICIs and slow tumor growth [152,153].

#### 5.4.4. Immunotherapy

Treating lung cancer in patients with ILD is notably difficult. While the standard of care in patients with advanced or metastatic NSCLC is a combination of a platinum-based doublet with immunotherapy [110], patients with ILDs are faced with skepticism in the fear of adverse events. Immunotherapy refers to immune checkpoint inhibitors (ICIs) and includes PD-1 inhibitors (such as nivolumab and pembrolizumab) and PD-L1 inhibitors such as atezolizumab and durvalumab). Adverse events can occur at any time in any organ [154]. Particularly, ICI-related pneumonitis, which in the context of IPF could be devastating due to their altered lung function, is the reason why patients with IPF are excluded from the majority of the studies. However, in patients without ILDs and NSCLs, immune-related adverse effects correlate to better outcomes [155]. Patients with ILD and lung cancer have similar levels of PD-L1 compared to the ones without ILD and increased tissue levels of PD-L1 are associated with better outcomes [156]. Other studies, however, report an increased expression of PD-1 and decreased expression of PD-L1 in mediastinal lymph nodes of patients with IPF compared to patients with lung cancer. The same expression pattern was observed in the lungs of patients with IPF compared to controls [83]. Mediastinal lymph nodes of bleomycin-treated mice showed increased size and higher PD-1 and PD-L1 mRNA levels than controls, while pembrolizumab attenuated bleomycin-induced fibrosis [83].

Monotherapy with ICIs for patients with NSCLC without ILD has an incidence of pneumonitis between 3–6%, which rises to 10% when ICIs are combined [157]. The incidence of pneumonitis in IPF is estimated to be between 7.3% and 42.9%, according to data from retrospective studies, including patients with NSCLC and IPF or ILA [158]. As was shown in a retrospective study with 123 patients with NSCLC treated with nivolumab and pembrolizumab, when the fibrosis score, defined visually in CT-scan according to the classification of Kazerooni et al. [159], increased from zero to >/1, the incidence of pneumonitis increased from 5.8% to 35.1% [160]. Increased fibrosis score and ground glass opacities are reported as risk factors for developing ICI-related pneumonitis in some studies [160,161,162]. A multicenter retrospective study from Japan, which included 200 patients with chronic interstitial pneumonia who were treated with ICIs, showed pneumonitis grades 3–5 in 15.5% and death in 4.5% of the patients. Pneumonitis manifested radiologically preferentially as organizing pneumonia (OP) (47.5%), while diffuse alveolar damage (DAD) was reported in more than 30% of the patients; DAD was associated with significantly worse survival compared to other histological patterns. Almost half of the patients suffered immune-related adverse events. Patients with adverse effects had a substantially better prognosis than those without, with a median PFS of 200 days versus 77 days and a median OS of 597 days versus 390 days. The objective response and disease control rates were 41.3% and 68.5%, respectively [163]. A meta-analysis of 179 patients in 10 studies with pre-existing ILD treated with ICIs for NSCLC showed that compared to patients without ILD, patients with pre-existing ILD had a substantially higher (almost double) overall response rate. The disease-control rate and progression-free survival in patients with preexisting ILD were similar to those without ILD. Patients with pre-existing ILD and NSCLC had a significantly higher incidence rate of any grade and grade 3 or higher CIP than those with NSCLC and without ILD (OR, 3.23; OR, 2.91) [164].

Case reports and a pilot study where nivolumab was administered as second-line therapy to patients with lung cancer and concomitant IPF after first-line chemotherapy failed suggested that nivolumab might be safely used in these patients. No signs of pneumonitis were observed during 12 weeks of treatment and efficacy showed partial response or stable disease [165,166]. A multicenter open-label single-arm phase II trial followed to evaluate the effectiveness and safety of nivolumab in patients with mild IPF and NSCLC: grade 2 pneumonitis was seen in 11% of the patients. The six-month PFS rate was 56%, the response rate was 39%, and the disease control rate was 72% [167]. These results are corroborated by another retrospective study with 461 patients (412 without ILD and 49 with ILD) who were treated with nivolumab or pembrolizumab, which showed that despite the increased frequency of pneumonitis, the response rate (RR), the disease control rate (DCR), the progression-free survival (PFS), and the overall survival (OS) were non-inferior in the ILD-NSCLC group than the patients with NSCLC without pre-existing ILD [168].

Most of the published case reports and original studies included Asian populations. In these patients, an increased risk of AE-ILD and chemotherapy-associated toxicity, including EGFR and TKI treatment, has been reported [169]. Therefore, additional studies in populations of other ethnic origins are helpful for comparison and extrapolation of the previously reported results. In a retrospective French study of 10,452 patients with advanced NSCLC who were treated with nivolumab, the effect of immunotherapy was examined in 148 patients (1.4%) with ILD. The overall survival time and the time-to-treatment duration were similar between patients with and without ILDs [170]. Survival was comparable in patients with idiopathic ILD, autoimmune/granulomatous ILD, and ILDs of other known causes. However, the results should be interpreted cautiously due to the small numbers in the different groups [170].

Although hesitancy to use immunotherapy in this group of patients is explained by the minimal, mostly retrospective research into the field, ICIs can be used in this population after assessing risks and benefits, so that therapy is tailored to the individual patient.

Treatment options for patients with NSCLC and ILDs are summarized in Table 4 and Table 5.

## 6. Treatment Options for SCLC Patients with ILD

SCLC is less common than NSCLC in patients with ILDs, accounting for approximately 20% of lung cancers [173]. Most reports suggest that patients with SCLC and ILD, particularly IPF, have reduced overall survival [174,175,176,177]. Individuals diagnosed with SCLC and ILD exhibit similar responses to standard treatment to the general population, provided they tolerate chemotherapy [174,175,178]. Pneumonitis was more often seen in patients with ILD and was associated with worse survival [175,179].

A phase II study of 33 participants with unresectable SCLC and IPF examined the effect of carboplatin, etoposide, and nintedanib combination: the incidence of AE-IPF after chemotherapy was 3% with an ORR of 68.8%, PFS of 4.2 months, and overall survival of 13.4 months, suggesting a role for nintedanib in preventing AE-IPF due to chemotherapy [180].

Immunotherapy in combination with chemotherapy has become the standard of care in extensive SCLC. Immunotherapy-induced interstitial lung disease incidence in large clinical trials is between 2.6 and 4%. Other studies have reported an incidence in Japanese patients up to 15.7%. Interestingly, the onset of immunotherapy-induced pneumonitis correlated to the presence of ILAs. These results suggest that the combination of chemotherapy and immunotherapy in patients with ILDs and SCLC should be used with extreme caution and an individualized approach should be practiced [181].

## 7. Palliative Care

Patients with fibrotic ILDs, and particularly IPF, have a dismal prognosis. The combination of fibrotic ILDs with lung cancer is a difficult situation for the patient, as well as for the treating physician. Impaired lung function, the risk of AE and pneumonitis, and comorbidities make many patients unfit or too frail for the usual therapeutic choices in the general population. The results of the DIAMORFOSIS study underline this problem [70]. Palliative treatment refers not only to pre-mortal interventions. Palliative treatment can greatly help alleviate patient’s symptoms in an early stage, particularly dyspnea, cough, and pain. Therefore, open discussion with the patients and their families is of paramount importance for improving their quality of life.

## 8. Conclusions

The relationship between lung cancer and ILD underscores the complexity of treatment decisions and potential complications. Retrospective studies have revealed varying rates of pneumonitis, including AE-ILD, in patients with ILD undergoing different therapeutic approaches for lung cancer. Factors such as the extent of ILD involvement, surgical resection, radiation dose, and specific chemotherapy agents have been identified as significant contributors to the mortality risk due to complications in these patients. These findings emphasize the need for careful consideration of treatment modalities and personalized approaches for individuals with both lung cancer and ILD. Future research and prospective studies are essential to refine treatment guidelines and enhance our understanding of the optimal management strategies for this complex patient population.

## Figures and Tables

**Table 1:** Main pathologic mechanisms contributing to the development of lung cancer and pulmonary fibrosis based on refs. [2,6,10,16,17,18,19,21,23,25,26,27,28,29,30,31,33,34,35,37,38,39,40,41]. For further explanation, see text.

Smoking Genetic susceptibility 2nd hit injury Chronic inflammation Aging	Gene mutations	P53 Surfactant protein genes JAK Fragile histidine triads Microsatellite instability Telomere shortening and expression (TERT, TERC) MET
	Signaling pathways	Tyrosine kinase signaling Hypermethylation of the CD90/Thy-1 promoter PD-1/PDL-1 MET upregulation
	Epithelial-mesenchymal transition	TGF-β Matrix metalloproteases
	Cell migration and invasion	Laminin Heat shock protein 27 Fasciin Matrix metalloproteases Integrins Intercellular channels formed by connexins (Cxs)
	Cellular senescence	Senescence-associated secretory phenotype (SASP)

P53: cellular tumor antigen p53; JAK: Janus kinase; TERT: telomerase reverse transcriptase; TERC: telomerase RNA component; MET: mesenchymal–epithelial transforming factor proto-oncogene; CD90/Thy-1: cluster of differentiation 90/Thymocyte differentiation antigen-1; PD-1: programmed cell death protein 1; PDL-1: programmed death ligand-1; TGF-β: transforming growth factor β.

**Table 3:** Challenges of different modalities for the diagnosis of lung cancer in patients with ILDs based on refs. [14,52,70,82,84,85,86,87,88,89].

Modality	Diagnostic Challenges
Pulmonary function tests (PFTs)	In CPFE, preserved lung volumes in patients may overestimate patients’ functional operability. DLCO is the most sensitive parameter
HRCT	Tumors may be directly adjacent to fibrotic lesions, with an underestimation of tumor size. Reduced sensitivity and specificity in evaluating mediastinal lymph nodes (reactive mediastinal lymph node enlargement may be seen in ILDs without lung cancer)
PET-scan	FDG-positive mediastinal lymph nodes may be reactive and not due to lung cancer infiltration
CT-guided biopsy	Motion artifacts and biopsy of fibrotic lesions adjacent to the tumor may give inconclusive results. Pneumothorax may be more difficult to treat.
Bronchoscopy with biopsy	Risk of acute exacerbation of ILD (AE-ILD). Tumor identification with radial EBUS or navigational bronchoscopy in fibrotic milieu may be more challenging, compared to patients without ILDs
Surgical biopsy	Increased risk of AE-ILD

CPFE: combined pulmonary fibrosis and emphysema; DLCO: diffusing capacity for carbon monoxide; HRCT: high-resolution computed tomography; PET: positron-emission tomography; ILD: interstitial lung disease.

**Table 4:** Proposed treatment for patients with NSCLC and ILDs based on refs. [14,52,171,172]. Decisions to be taken by the oncology board, considering the patient’s wishes, comorbidities, NSCLC stadium, and ILD stadium. Oncologic outcomes and preservation of lung function to be considered. Close monitoring for AE-ILD and pneumonitis required.

Stage of NSCLC	Treatment Options for NSCLC with ILD
Stage I	- Surgical resection (lobectomy or segmentectomy): for patients with stable and well-controlled ILD, with mild to moderately impaired lung function - Stereotactic body radiation therapy (SBRT): alternative to surgery for patients who are not surgical candidates due to their ILD, due to poor lung function or comorbidities. Option in a minority of very carefully selected patients due to the possible detrimental risk of pneumonitis - Percutaneous image-guided ablation to be considered in patients with poor lung function and small tumors
Stage II	- Surgical resection: for patients with stable and well-controlled ILD, with mild to medium impaired lung function - SBRT: For patients unsuitable for surgery due to ILD, SBRT may be considered. Option in a minority of very carefully selected patients due to the possible detrimental risk of pneumonitis
Stage III	- Radio chemotherapy: standard of treatment for locally advanced NSCLC. Decision to proceed in patients with ILD to be taken in oncology board, considering the high potential risk of pulmonary toxicity. Sequential, instead of concomitant therapy may be associated with reduced risk of pulmonary toxicity. Radiation therapy may be an option in a small minority of very carefully selected patients due to the possible detrimental risk of pneumonitis - Immunotherapy: may be considered in patients with stable ILD and mild to moderately impaired lung function, as part of the treatment regimen
Stage IV	- Systemic therapy: chemotherapy, targeted therapy, immunotherapy, or a combination of the above depending on the patient’s performance status, ILD status and comorbidities - Palliative care: in advanced stages of NSCLC or end-stage ILD

**Table 5:** Systemic treatment considerations for patients with ILD and advanced (stage IV) NSCLC, based on refs. [171,172].

Treatment	Indication	Special Considerations in Patients with ILDs
TARGETED THERAPIES		
EGFR-TKI (e.g., afatinib, erlotinib, dacomitinib, gefitinib, osimertinib) ALK inhibitors (e.g., alectinib, brigatinib, ceritinib, crizotinib, lorlatinib) ROS1 Inhibitors (e.g., ceritinib, crizotinib, entrectinib) BRAF Kinase Inhibitors (e.g., dabrafenib, vemurafenib)	EGFR Exon 19 deletion or Exon 21 L858R ALK rearrangement ROS1 rearrangement BRAF V600E mutation	High incidence of pneumonitis, particularly in smokers, patients with pre-existing ILD of Asian origin Gefitinib particularly high risk of pneumonitis Combination with anti-VEGF may reduce risk of pneumonitis Very close monitoring required Suggestion for non-administration by the Japanese Thoracic Society with very low strength of the recommendation
IMMUNE CHECKPOINT INHIBITORS		
PD-1/PDL-1 Inhibitors (e.g., nivolumab, pembrolizumab, atezolizumab, durvalumab, avelumab, cemiplimab) CTLA-4 Inhibitors (e.g., ipilimumab, tremelimumab)	First line for NSCLC as monotherapy (pembrolizumab, atezolizumab, cemiplimab) or in combination with platin-based regiments according to PDL-1 expression Continuation maintenance Second line depending on first line treatment	High incidence of pneumonitis, particularly in smokers with pre-existing ILD Combination with anti-VEGF agents may reduce risk of pneumonitis CTLA-4 inhibitors may be safer compared to PD-1/PDL-1 inhibitors regarding the risk of pneumonitis Very close monitoring required Suggestion for non-administration by the Japanese Thoracic Society; however, this therapy may be a reasonable option in some patients (very low strength of the recommendation)
CHEMOTHERAPY		
Carboplatin or cisplatin combination therapy Combination options include: Docetaxel Etoposide Gemcitabine Paclitaxel Pemetrexed	First line for NSCLC in patients with contraindications to ICIs	Cis- and carboplatin considered the safest option for patients with ILDs Docetaxel associated with increased risk of AE-ILD, not recommended Combination with platinum considered safe Gemcitabine associated with increased risk of AE-ILD, not recommended Combination of paclitaxel with platinum one of the safest combinations Pemetrexed associated with increased risk of AE-ILD Suggestion for administration of cytotoxic drugs by the Japanese Thoracic Society; however, this therapy may not be a reasonable option in some patients (low strength of the recommendation)
ANTI-VEGF AGENTS		
Bevacizumab Nintedanib Ramucirumab	First line non-squamous Second line for adenocarcinoma in combination with docetaxel Second line NSCLC	Bevacizumab reported to prevent chemotherapy-induced AE-ILD in patients with ILD and NSCLC ORR significantly improved for nintedanib plus chemotherapy vs. chemotherapy in patients with nonsquamous histology. Overall survival improved only in patients with nonsquamous histology and patients with GAP stage I (J-Sonic trial) Nintedanib is approved for IPF, SScl-associated ILD and progressive pulmonary fibrosis Anti-VEGF agents may reduce risk of pneumonitis due to TKIs and ICIs

EGFR: epidermal growth factor receptor; TKI: tyrosine kinase inhibitor; Exon 21 L858R: exon 21 L858 substitution; ALK: anaplastic lymphoma kinase; ROS1: ROS-proto-oncogene 1, receptor tyrosine kinase; BRAF: B-Raf proto-oncogene, serine/threonine kinase; VEGF: vascular endothelial growth factor; PD-1: programmed cell death protein-1; PD-L1: programmed death-ligand 1; CTLA-4: cytotoxic T-lymphocyte-associated protein-4.
